# Construction and *in vitro* evaluation of a recombinant live attenuated PRRSV expressing GM-CSF

**DOI:** 10.1186/s12985-014-0201-4

**Published:** 2014-11-25

**Authors:** Lingxue Yu, Yanjun Zhou, Yifeng Jiang, Wu Tong, Shen Yang, Fei Gao, Kang Wang, Liwei Li, Tianqi Xia, Qun Cheng, Guangzhi Tong

**Affiliations:** Shanghai Veterinary Research Institute, Chinese Academy of Agricultural Sciences, No. 518, Ziyue Road, Minhang District Shanghai, 200241 China; Jiangsu Co-innovation Center for Prevention and Control of Important Animal Infectious Diseases and Zoonoses, Yangzhou, 225009 China

**Keywords:** PRRSV, GM-CSF, BMDCs

## Abstract

**Background:**

Porcine reproductive and respiratory syndrome virus (PRRSV) continues to be an important problem for the swine industry. Inactivated vaccines and modified-live virus vaccines are widely used in the field; however, the efficacy of these PRRSV vaccines is suboptimal due to poor immunogenicity. Granulocyte–macrophage colony stimulating factor (GM-CSF) has been extensively used as an effective genetic and protein adjuvant to enhance the efficiencies vaccines expressing tumor or pathogen antigens. The purpose of this study was to determine if GM-CSF could increase the efficiency of PRRSV vaccine.

**Methods:**

The GM-CSF gene was inserted in the HuN4-F112 vaccine strain by overlap PCR. The expression of GM-CSF by the recombinant virus was confirmed with methods of indirect immunofluorescent assay (IFA) and Western blotting. The stability of recombinant virus was assessed by cDNA sequence and IFA after 20 passages. To detect the biological activity of GM-CSF expressed by the recombinant virus, bone marrow-derived dendritic cells (BMDCs) were isolated and co-cultured with the recombinant virus or parental virus and the surface phenotypes of BMDCs were examined by flow cytometric analysis. The cytokines secreted by BMDCs infected with PRRSV, or treated with LPS, GM-CSF or medium alone were evaluated by ProcartaPlexTM Multiplex Immunoassays and qRT-PCR.

**Results:**

A novel modified-live PRRSV vaccine strain expressing GM-CSF (rHuN4-GM-CSF) was successfully constructed and rescued. The GM-CSF protein was stable expressed in recombinant virus-infected cells after 20 passages. Analysis of virus replication kinetics showed that the novel vaccine strain expressing GM-CSF had a similar replication rate as the parental virus. *In vitro* studies showed that infection of porcine BMDCs with rHuN4-GM-CSF resulted in increased surface expression of MHCI+, MHCII + and CD80/86+ that was dependent on virus expressed GM-CSF. The expression of representative cytokines was significantly up-regulated when BMDCs were incubated with the recombinant GM-CSF expressing virus.

**Conclusions:**

Our results indicated that the expression of GM-CSF during infection with a vaccine strain could enhance the activation of BMDCs and increase cytokine response, which is expected to result in higher immune responses and may improve vaccine efficacy against PRRSV infection.

## Background

Porcine reproductive and respiratory syndrome (PRRS) is a respiratory disease of pigs, and infects porcine of all ages. PRRS causes reproductive failure in breeding swine. PRRS was first described in North America in 1987 and become responsible for major economic losses to the swine industry [1]. The causative agent of PRRS is PRRS virus (PRRSV), which is an enveloped positive-strand RNA virus and belongs to the family of *Arterivirida*e [2]. PRRSV is monocytotropic, which directly infects subsets of macrophages and DCs and subverts overall immune responses [3].

In 2006, a highly virulent strain of PRRSV (HP-PRRSV), which caused significant morbidity can be characterized by high fever, was first detected in China and then rapidly spread to most provinces of China [4-6]. Under these circumstances, vaccination against PRRSV provides invaluable support to increase host resistance, reduce environmental contamination, and reduce the chance of regional outbreaks. Two types of PRRSV vaccines are now available commercially: an inactivated vaccine and the modified-live virus vaccine. Several live attenuated North American-lineage PRRSV vaccines, such as Ingelvac1 ATP, RespPRRS MLV, RespPRRS/Repro1 ATP, and CH-1R, have been successfully employed in field [7], and induce higher protection rates than with inactivated vaccines. To respond to the recently emerged HP-PRRSV, new live attenuated vaccines were developed by passaging HP-PRRSV isolates in MARC-145 cell lines [7-9].

The currently available modified live vaccines have shown the most promising efficacy, however, there are imperative issues that need to be addressed. Some examples are the partial protection against heterologous viruses [10], delayed and suboptimal cell-mediated immunity (CMI) and neutralizing antibody levels after vaccination [11-13].

In an effort to develop more efficacious vaccines that induced broader immune responses and protection against heterologous PRRSV strains, live virus-vectored vaccines were made by expressing immune response-specific molecules [14,15]. Recruitment and activation of dendritic cells (DCs) plays an important role in both innate and adaptive immune responses to viral infections, including vaccination with live attenuated vaccines [14,16]. GM-CSF as one of the most important cytokines, has been used to differentiate DCs from monocytes and bone marrow cells [17], as well as to mature and/or activate DCs *in vitro* and *in vivo* [14,18,19]. Therefore, GM-CSF has been widely employed as an attractive adjuvant to apply in therapeutic treatment and in vaccine formulations [14,16,20-25]. In this study, we constructed a recombinant PRRSV expressing GM-CSF, and provide evidence that this recombinant vaccine effectively activated DCs and regulate the anti-virus microenvironment *in vitro*.

## Results

### Construction and rescue of the recombinant PRRSV

**Figure 1:**
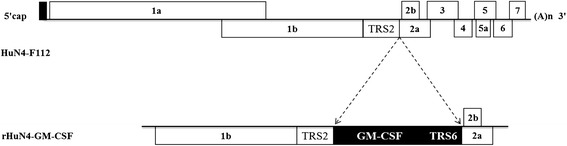
**Construction of the plasmid HuN4-GM-CSF.** Porcine GM-CSF and a copy of the transcription regulatory sequence for PRRSV ORF6 (TRS6) were inserted between ORF1b and ORF2 of the infectious molecular clone of the HuN4-F112 vaccine strain by overlap PCR.

**Figure 2:**
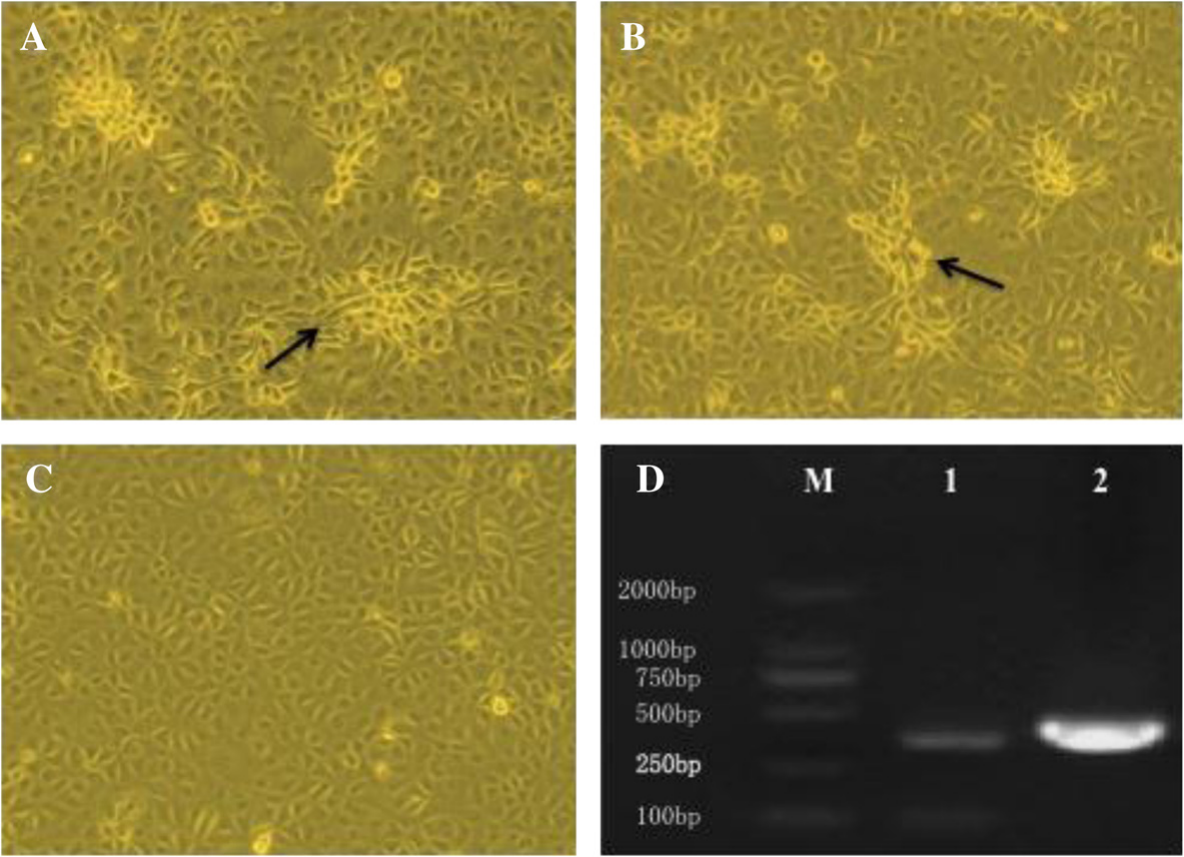
**Rescue of the recombinant PRRSV.** Cytopathic effects (CPE) were observed on MARC-145 cells after infection by viruses for 3 days. CPE was observed in rHuN4-GM-CSF **(A)** and HuN4-F112 **(B)**, while mock-infected cells remained normal **(C)** Original Magnification 100×. **(D)** Identification of the recombinant virus and parental virus with genomic marker. The presence of a *Mlu* I restriction site in recombinant virus resulted in fragments of 360 bp and 92 bp. (1) rHuN4-GM-CSF; The RT-PCR fragments derived from the parental isolate was not digested by Mlu I (2) HuN4-F112; (M) Marker DL2000.

Pei et al [26] and Lawson SR et al [15] showed that the position between ORF1b and ORF2 of PRRSV is a suitable site for foreign gene insertion [15,26]. In this study, the GM-CSF gene and the synthesized TRS6 sequence were inserted between the stop codon of ORF1b and the start codon of ORF2 of the infectious molecular clone of HuN4-F112 vaccine strain by overlap PCR (Figure 1). The full-length cDNA clone pHuN4-GM-CSF was verified by nucleotide sequencing (as described in Materials and Methods). The recombinant virus rHuN4-GM-CSF was rescued by transfecting BHK-21 cells (Baby hamster Kidney cells clone 21) with plasmid expressing the recombinant virus and passaging of the supernatants in MARC-145 cells. Cytopathic effects (CPE) were observed on MARC-145 cells 3 days post-infection (Figure 2A to C). As shown in Figure 2D, the RT-PCR fragment derived from the recombinant virus was cleaved by restriction enzyme *Mlu* I, generating two fragments of 360 bp and 92 bp (lane 1). In contrast, no cleaved fragment was detected in the PCR amplification products derived from the parental isolate virus under the digestion of *Mlu* I (lane 2). Sequence determination of inserted GM-CSF gene confirmed that the recombinant virus rHuN4-GM-CSF was successfully generated (data not shown).

### Expression of GM-CSF by the recombinant virus

**Figure 3:**
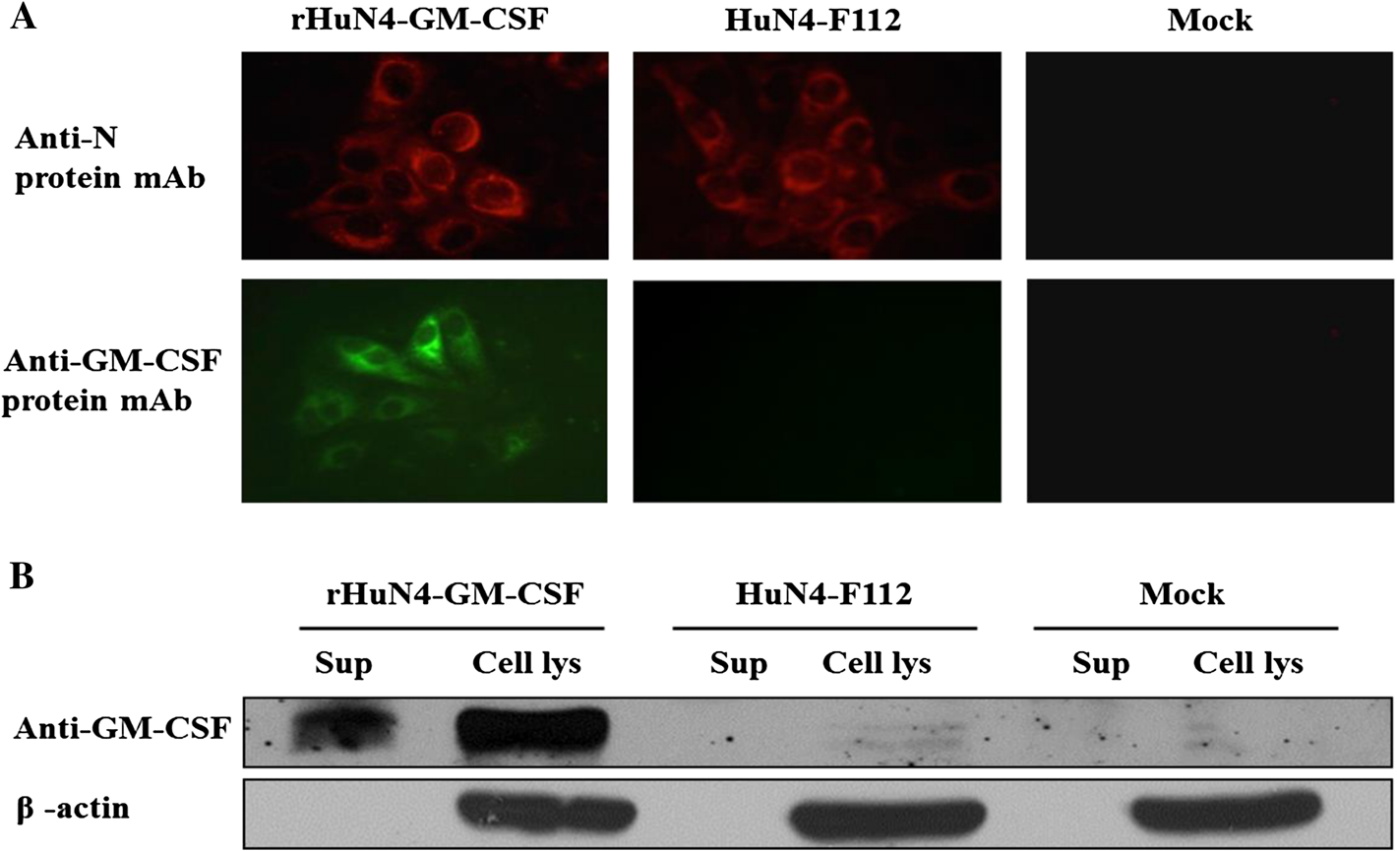
**Expression of GM-CSF by rHuN4-GM-CSF.** Cells were infected at a multiplicity of infection (MOI) of 1 with viruses and incubated for 24 hours before analysis. **(A)** The virus-infected MARC-145 cells were fixed and tested by IFA to determine the expression of PRRSV N protein and porcine GM-CSF. Original Magnification 200×. **(B)** Western blot detection of GM-CSF in the supernatant fraction (sup) or cell lysates (cell lys) of MARC-145 cells infected with viruses.

We next investigated whether the inserted GM-CSF gene could be expressed in recombinant PRRSV-infected cells. As expected, mouse anti-GM-CSF monoclonal antibody (mAb) specifically detected the MARC-145 cells infected with rHuN4-GM-CSF in IFA (Figure 3A), but not cells infected with the HuN4-F112 control virus. As shown in Figure 3B, the expression of GM-CSF protein (22 kDa) was detected in the supernatant and cell lysates of MARC-145 cells that infected with the recombinant rHuN4-GM-CSF, but not the parental virus, by Western blotting using anti-porcine GM-CSF mAb.

### Characterization of the recombinant PRRSV expressing the GM-CSF gene

**Figure 4:**
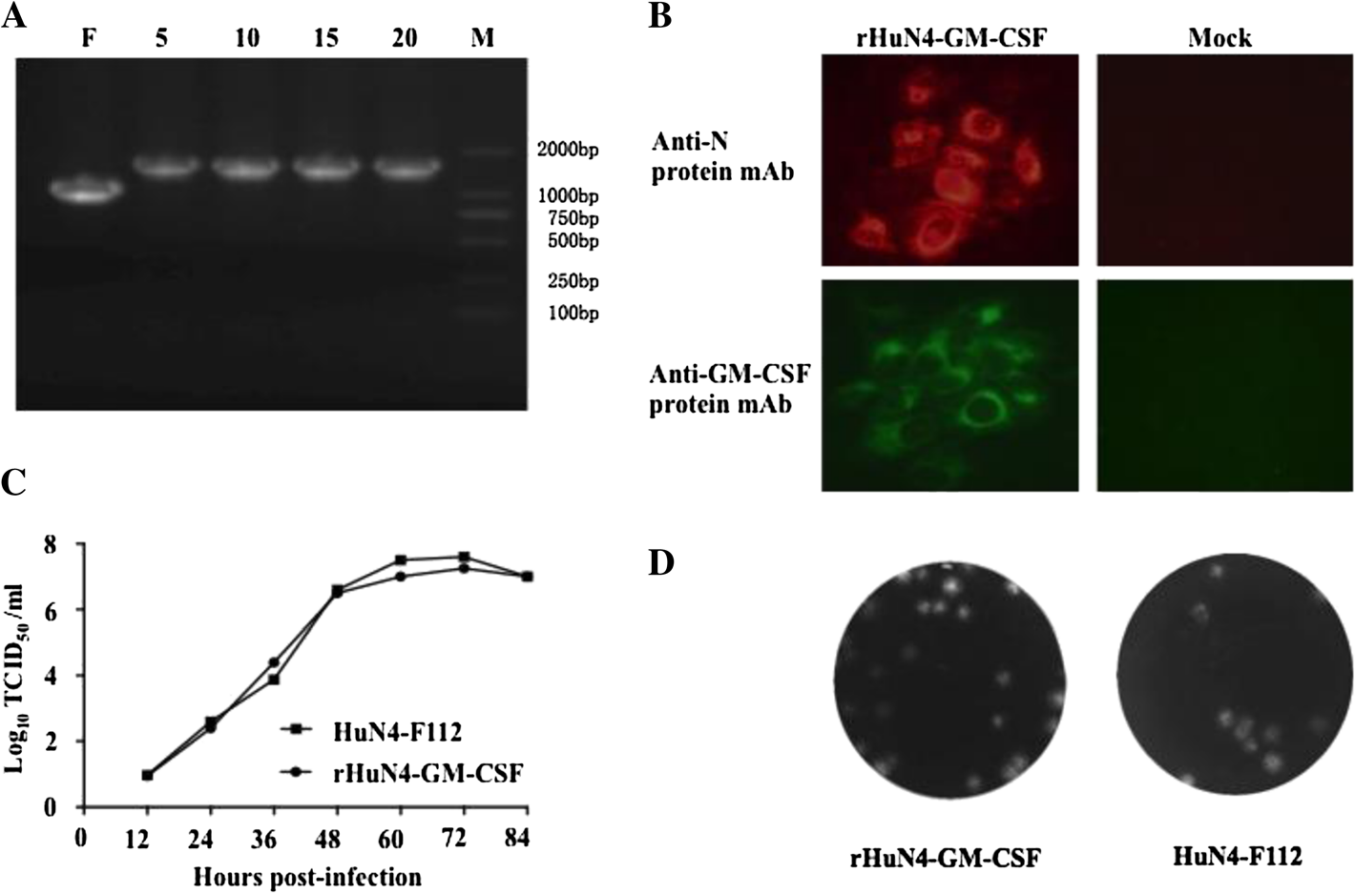
**Characterization of the recombinant PRRSV expressing the GM-CSF gene in MARC-145 cells. (A)** Detection of the GM-CSF gene insertion in the recombinant virus. F was product of the parental virus; the numbers show the passages of the recombinant viruses; M was Marker DL2000. The recombinant viruses used in **B**, **C** and **D** were passaged in MARC-145 cells for 20 passages. **(B)** Detection of GM-CSF protein expression in recombinant virus-infected MARC-145 cells by IFA. Original Magnification 200×. **(C)** Growth kinetics comparison between rHuN4-GM-CSF and the parental virus in MARC-145 cells. The infection was done as mentioned above. The cell supernatants were harvested at the indicated time points and titrated in MARC-145 cells. **(D)** Plaque assays for the parental virus and rHuN4-GM-CSF. MARC-145 cells infected with viruses were stained with crystal violet at 4 days post infection.

To investigate whether the inserted gene could be stably maintained in the recombinant virus, we serially passaged the virus on MARC-145 cells to 20 times. The GM-CSF gene encoded by the recombinant PRRSV was detected by PCR amplification (Figure 4A) and confirmed by Sanger sequencing (data not shown). Consistent with the previous IFA data, the expression of GM-CSF by MARC-145 cells infected with the recombinant virus could be detected by IFA after 20 passages (Figure 4B).

The viral replication kinetics of the recombinant virus and the parental control were examined in MARC-145 cells to determine if the insertion of the GM-CSF sequence or if GM-CSF expression by virus-infected cells had an impact on viral replication. The supernatants from cells infected with recombinant virus or parental virus was collected at 12, 24, 36, 48, 60, 72 and 84 hpi for virus titration. As evidenced by the viral titers, the replication kinetics of the recombinant rHuN4-GM-CSF was very similar to that of the parental virus (Figure 4C). The plaque morphology and size of rHuN4-GM-CSF on MARC-145 cells were also similar to that of the parental virus (Figure 4D). These results indicated that the insertion or expression of the GM-CSF gene did not affect the growth rate of the HuN4-F112 vaccine strain.

### Maturation and activation of BMDCs stimulated by rHuN4-GM-CSF

**Figure 5:**
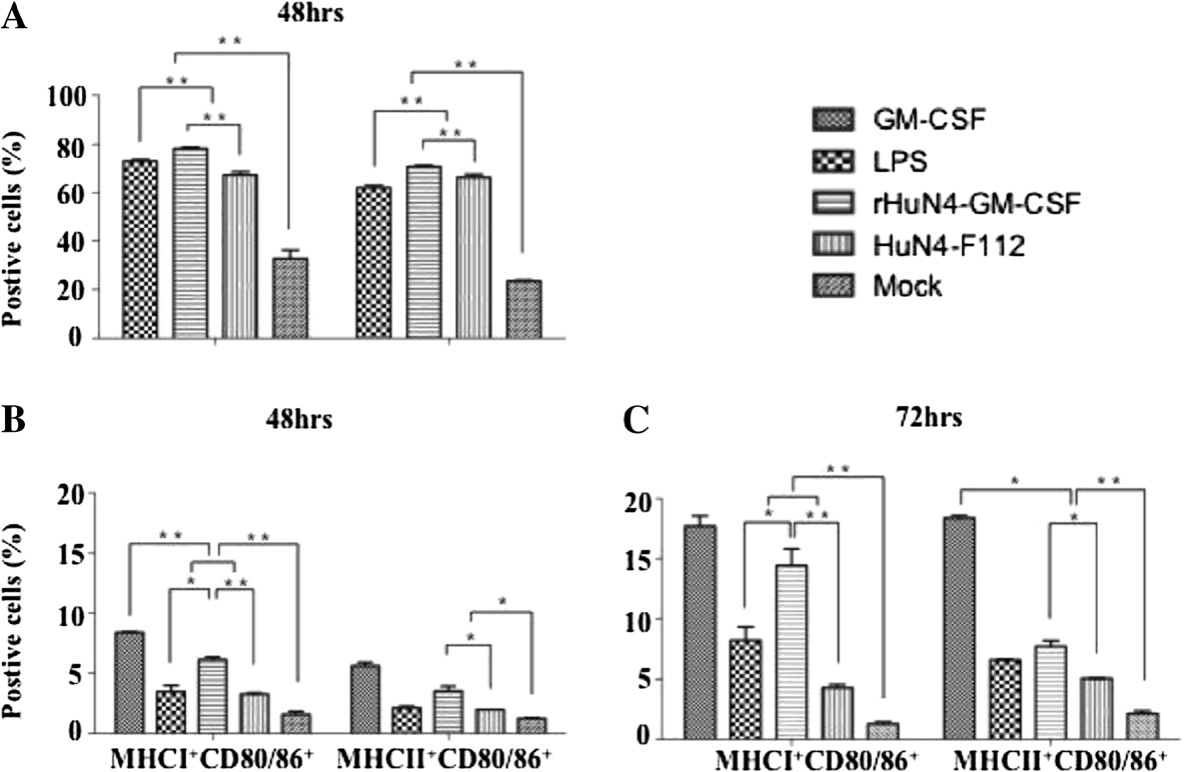
**Maturation and activation of BMDCs stimulated by rHuN4-GM-CSF.** BMHCs as DC precursors were cultured with or without GM-CSF. The cells were infected with viruses. LPS and GM-CSF were used as a positive control, and the medium from untreated cells (Mock) served as a negative control. Maturation of BMDCs treated by GM-CSF and then inoculated virus at 48 hpi **(A)**. Activation of BMDCs were cultured without GM-CSF, but directly infected with viruses at 48 hpi **(B)** and 72 hpi **(C)**. All data are the means from three independent experiments with cells from different donors. The horizontal lines represent the geometric mean for each group, and statistical analysis was performed. *: P < 0.05; **: P < 0.01.

To test the biological activity of GM-CSF expressed by the recombinant virus, BMDCs were isolated from porcine bone marrow, and co-cultured with the recombinant virus or parental virus. BMDCs treated with lipopolysaccharide (LPS) were included as a positive control. BMDCs were infected with PRRSV at MOI of 0.03 and the surface phenotypes of these cells were examined by flow cytometric analysis at 48 hpi. As shown in Figure 5A, infection with rHuN4-GM-CSF promoted better maturation and/or activation of BMDCs than infection with the parental virus, when they were pretreated with GM-CSF, as shown by significantly more MHC I^+^/CD 80/86^+^ and MHC II^+^/CD 80/86^+^ double positive cells.

On the other hand, BMDCs without prior treatment of GM-CSF were incubated with PRRSV at MOI of 0.3 and the surface phenotype expressions of MHC I, MHC II and CD 80/86 were evaluated by flow cytometry at 48 and 72 hpi. LPS and GM-CSF individually were as positive control. As expected, rHuN4-GM-CSF induced more MHC I^+^/CD 80/86^+^ and MHC II^+^/CD 80/86^+^ doubly positive cells than the parental virus (Figure 5B and C). As a negative control, very few GM-CSF-treated or non-treated BMDCs were activated by the medium alone. These results indicated that the rHuN4-GM-CSF expressing GM-CSF promoted differentiation of myeloid progenitor cells to DCs *in vitro*.

### Cytokines secreted by BMDCs treated with rHuN4-GM-CSF

**Figure 6:**
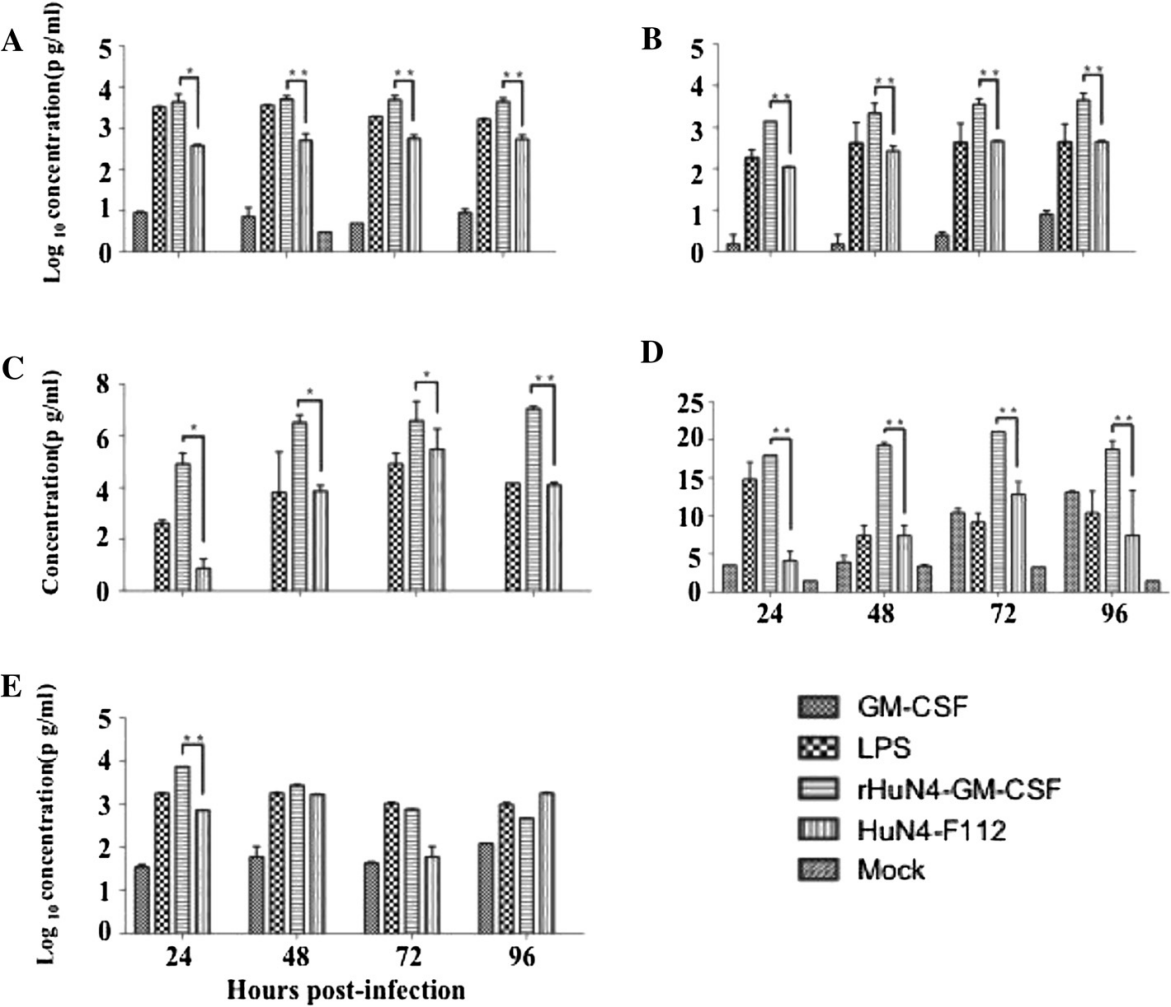
**Cytokines secreted by BMDCs treated with rHuN4-GM-CSF.** The expression of cytokines determined by ProcartaPlexTM Multiplex Immunoassays. BMDCs were incubated with either rHuN4-GM-CSF or the parental virus at MOI of 0.3 at 24, 48, 72 and 96 hpi. LPS and GM-CSF were used as a positive control, and the medium from untreated cells (Mock) served as a negative control. **(A)** IL-1β; **(B)** IL-12; **(C)** IL-4; **(D)** IFN-γ; **(E)** IFN-α. The horizontal lines represent the geometric mean for each group, and statistical analysis was performed. *: P < 0.05; **: P < 0.01.

The cytokines secreted by BMDCs infected with PRRSV, or treated with LPS, GM-CSF or medium alone were as evaluated by ProcartaPlex™ Multiplex Immunoassays. As shown in Figure 6A, B and D, a significantly higher level of IL-1β, IL-12 and IFN-γ were observed for rHuN4-GM-CSF infected BMDCs compared to that of cells infected with the parental virus from 24 to 96 hpi. Despite the low levels, we detected slightly higher expression of IL-4 and TNF-α in BMDC treated by rHuN4-GM-CSF than treated by the parental virus (Figure 6C and E).

**Figure 7:**
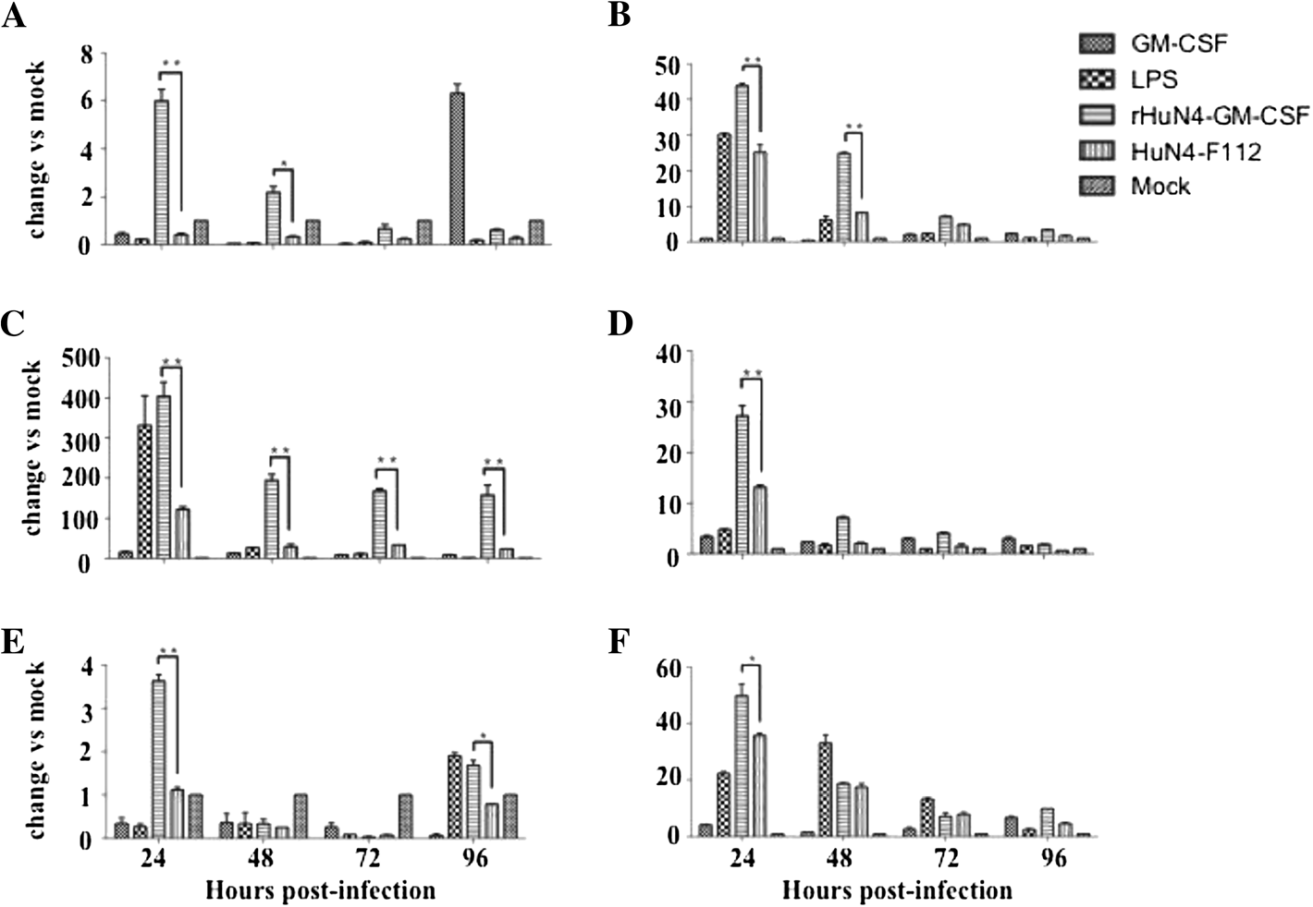
**Quantification of cytokines of BMDCs by qRT-PCR.** BMDCs were incubated with either rHuN4-GM-CSF or the parental virus at MOI of 0.3 at 24, 48, 72 and 96 hpi. LPS was used as a positive control, and the medium from untreated cells (Mock) served as a negative control. Total RNA was prepared and used in a qRT-PCR to determine levels of mRNA levels for cytokines. **(A)** GM-CSF; **(B)** IL-12; **(C)** IL-1β; **(D)** IFN-γ; **(E)** IL-4; **(F)** IFN-α. The horizontal lines represent the geometric mean for each group, and statistical analysis was performed. *: P < 0.05; **: P < 0.01.

In addition, qRT-PCR was performed to measure mRNA expression level of GM-CSF, IL-1β, IL-4, IL-12, IFN-γ and TNF-α at 24, 48, 72 and 96 hpi. As shown in Figure 7, the mRNA expression level of GM-CSF was significantly higher in rHuN4-GM-CSF infected BMDCs than that of cells infected with the parental virus until 48 hpi (Figure 7A). The expression of IL-1β in BMDCs infected with rHuN4-GM-CSF was significantly higher than that infected with the parental virus until 96 hpi (Figure 7C). The expression of IL-12, IL-4 and IFN-γ was also higher than other groups at 24 hpi (Figure 7B, C and D). Our data suggested that the recombinant virus expressing GM-CSF, a DC-stimulating molecule, activated DCs and induced higher levels of cytokines expression.

## Discussion

PRRSV is one of the most economically significant viral pathogens for pig production worldwide. PRRSV infection could impair the ability of APCs to stimulate antigen-specific immune responses, dysregulate expression of various inflammatory cytokines, which could disrupt the establishment of an anti-virus microenvironment *in vivo*. Vaccines are widely used to control PRRSV; however, these vaccines can result in immunosuppression and confer limited heterologous protection [11-13]. As one of the most efficient adjuvants, GM-CSF has been widely studied to enhance the immunogenicity of tumor and pathogen antigens [14,16,20-25]. Here, we successfully generated a recombinant PRRSV virus expressing GM-CSF, rHuN4-GM-CSF, and demonstrated *in vitro* that rHuN4-GM-CSF has the potential to be a more efficient candidate vaccine.

Previous studies proved that PRRSV could tolerate the addition of foreign genes, which were fused to ORF1b, ORF7, or Nsp2 [27-31]. However, insertion of extra non-viral genes has reduced levels of replication or resulted in genetic instability such that the recombinant viruses no longer encode a functional foreign gene after serial passage. TRS has been reported to drive transcription of the inserted gene without affecting expression of virus genes in recombinant PRRSV [15,26,28]. Moreover, previous studies showed that GM-CSF is a safe and efficacious adjuvant for enhancing the viral specific immune response and has been investigated in several viral vectors, such as vesicular stomatitis virus (VSV) [25], retrovirus [20] and vaccinia virus (VV) [22,24]. Recently, the recombinant Rabies virus (RABV) expressing GM-CSF enhanced the immunogenicity of RABV via recruitment and activation of DCs and B cells in the draining lymph nodes [14,16]. Therefore, we generated the recombinant virus rHuN4-GM-CSF by inserting the GM-CSF between ORF1b and ORF2 using the original TRS2 to drive transcription of GM-CSF and inserting an extra synthetic TRS6 to drive transcription of the ORF2 genes. This recombinant virus showed similar replication ability as compared to the parent PRRSV vaccine strain. Furthermore, the recombinant virus stably expressed GM-CSF following 20 passages in MARC-145 cells.

It is known that GM-CSF regulates Th1 and Th2 immune responses by activation and maturation of DCs, DCs are the most efficient APCs and thus play a key role in both innate and adaptive immune responses *in vivo* [32]. Furthermore, mature and activated DCs are critical to the activation of T cells and virus-specific adaptive immune responses [33]. When porcine bone marrow hematopoietic cells (BMHCs) were stimulated with GM-CSF, three types of myeloid cells were generated: adherent macrophages, non-adherent granulocytic cells and BMDCs. The adherent macrophage cells expressed lower levels of both MHC II and CD80/86 compared to BMDCs. BMDCs, as the dominant cell type, were MHC II^high^ CD80/86^+^ whereas granulocytic cells were MHCII¯/^low^ [34,35]. MHC II molecules are expressed only on APCs. T cells that recognize only antigenic peptides displayed with MHC II generally function as T helper cells. In the study, as expected, rHuN4-GM-CSF induced more MHC II^+^/CD 80/86^+^ doubly positive cells than the parental virus (Figure 5) *in vitro*. Thus, mature and activated DCs are critical to the activation of T cells and virus-specific adaptive immune responses. In addition, MHC I molecules are expressed on nearly all nucleated cells. The cell-mediated immune response to viral infections is the presentation of virus-encoded peptides by MHC I molecules to T cytotoxic cells [36]. BMDCs could be infected by PRRSV [37]. In the study, results show that rHuN4-GM-CSF induced more MHC I^+^/CD 80/86^+^ doubly positive cells than the parental virus (Figure 5). The up-regulated expression of MHC I molecules of BMDCs allows the virus to evade detection and destruction by cytotoxic T-cells*.* Furthermore, the recombinant rHuN4-GM-CSF vaccine would trigger stronger cell-mediated immune response to viral infections than the parental HuN4-F112 vaccine *in vivo*.

Our results demonstrated that higher levels of IL-12, IL-1β, IL-4 (Th2) and IFN-γ (Th1) were produced following infection of BMDCs with the recombinant virus than with the control virus. Previous studies proved that Th1 responses are characterized by the development of antigen-specific IFN-γ-secreting T cells [38] and IFN-γ promotes co-stimulatory molecule and pro-inflammatory cytokines production (such as IL-1 and IL-12) [39,40]. Of particular interest, IL-12 is a heterodimeric cytokine that is produced mainly by activated myeloid DCs and plays a pivotal role in the differentiation and expansion of Th1 cells [41,42]. In this study, the recombinant virus could induce the higher mRNA and protein levels of IL-12 in BMDCs, compared to the parent live-attenuated virus. High expression of IL-12 by DCs augments NK cells and T cells mediated cytotoxicity and stimulate production of IFN-γ and proliferation of NK cells and T cells as well [43-45]. The increased levels of cytokines production following rHuN4-GM-CSF virus infection would likely improve the ability of APCs to stimulate antigen-specific immune responses and regulate the anti-virus microenvironment. Base on this research, GM-CSF may be an effective adjuvant expressed by recombinant virus. DCs play a critical role in the development of both protective and pathogenic aspects of antiviral immunity. Upon the addition of IFN-γ, there would be maturation and activation of DCs and high-level release of IL-12[46,47]. Myeloid DCs are also target of Dengue virus and Measles virus. It would be used to develop new vaccines and therapeutic strategies by construction recombinant attenuated viruses expressing molecular adjuvants.

## Conclusions

In summary, the recombinant virus expressing GM-CSF was successfully constructed and rescued. The GM-CSF gene was stably expressed and did not affect the replication phenotype of the parent virus. Importantly, *in vitro* results showed that rHuN4-GM-CSF enhance the activation of DCs and promoted the secretion of several cytokines. These results demonstrated that the recombinant virus expressing GM-CSF holds great promise and is likely to be a more efficacious PRRSV candidate vaccine.

## Materials and methods

### Virus strains and cell lines

HuN4-F112, an attenuated vaccine virus strain adapted from the highly pathogenic PRRSV (HP-PRRSV) HuN4 strain (GenBank accession no. EF635006), was propagated and titrated in MARC-145 cells (African green monkey kidney cell line) [9]. BHK-21 cells were used to rescue virus by transfection with in vitro transcribed viral RNA.

### Construction of PRRSV expressing GM-CSF

Sequences that correspond to GenBank (U67175.1) and encode porcine GM-CSF protein were synthesized in our laboratory. The sequence encoding GM-CSF with signal peptide inserted between ORF1b and ORF2 in the infectious molecular clone of the HuN4-F112 vaccine strain by overlap PCR to generate the full-length reverse genetics clone, pHuN4-GM-CSF. The primers used for overlap PCR are described in Table 1. The expression of GM-CSF in the construct was controlled by the transcription-regulating sequence 2 (TRS2) for ORF2, and an extra synthetic transcription-regulating sequence 6 (TRS6) derived from ORF6 was inserted downstream of the GM-CSF gene to drive the expression of ORF2. Hence, GM-CSF is expressed from an independent subgenomic RNA.

**Table 1:** **Primers uesd for construction of recombinant PRRSV infectious clones**

**Primer**	**Nucleotide sequence (5′–3′)**	**Sense**	**Relative position in the genome**
**F1**	TTCCGGAGACAGTCTTCAGC	+	11143bp-11162bp
**R1**	TTCTGCAGCCACATTTCAATTCAGGCCTAAAGTT	-	11963bp-11982bp
**F2**	AACTTTAGGCCTGAATTGAAATGTGGCTGCAGAA	+	11963bp-11982bp
**R2**	TTGCATAGACCCCATTTCAT*CGTTCCGCTGAAACTCTGGTTAAAGGGGTTGCCGCGGAAC*TTACTTTTTGACTG	-	11983bp-12002bp
**F3**	GTTTCAGCGGAACGATGAAATGGGGTCTATGCAA	+	11983bp-12002bp
**R3**	GAAAGGCCTCATAAGATCTTCT	-	12196bp-12217bp

Bold letters (nucleotides) in sequences denote the restriction enzyme sites (F1-*BspE* I;R3-*Bgl* II), and the italics in the sequence indicate the transcription-regulating sequence 6 of PRRSV(TRS6).

To rescue the virus, capped RNAs of the viral genome produced by in vitro transcription reactions with the recombinant clone were transfected into BHK-21 cells [48]. The rescued recombinant viruses were propagated in MARC-145 cells, which were maintained in DMEM with 10% heat-inactivated fetal bovine serum (Life Technologies, Switzerland). The infected MARC-145 cells were monitored daily for the formation of cytopathic effect (CPE). Rescued recombinant virus RNAs were extracted from cell culture supernatants of the infected cells by using an RNeasy Plus Mini kit (QIAGEN). RT-PCR was performed as described previously. And the RT-PCR amplified product was digested by *Mlu* I to check the genetic marker engineered into the recombinant virus [48].

GM-CSF gene expression by the recombinant virus was confirmed by methods of indirect immunofluorescent assay (IFA) and Western blotting. For IFA, MARC-145 cells were infected with the virus at MOI of 1. The primary antibodies used were specific monoclonal antibodies against PRRSV nucleocapsid protein [49] and porcine GM-CSF (R&D systems, MN), respectively. The secondary antibodies were Alexa Fluor®488 donkey anti-mouse IgG (H + L)(For GM-CSF detection) and Alexa Fluor®568 goat anti-mouse IgG (H + L)(for N protein detection) (Life Technologies, Switzerland). To confirm synthesis of GM-CSF recombinant protein from rHuN4-GM-CSF in MARC-145 cells, the culture supernatant and cells were collected and analyzed by Western blotting. Monoclonal antibody against porcine GM-CSF and anti-β-actin antibody (Sigma-Aldrich) were used as primary antibodies. Primary antibody reactivity was visualized using a chemioluminescence detection system (Thermo Fisher Scientific).

### Confirming the inserted gene within the recombinant virus

To assess the stability of the insert in recombinant virus, the rescued virus was serially passaged in MARC-145 cells for twenty times. At the 5th, 10th, 15th, and 20th passages, the inserted gene was amplified by RT-PCR and the cDNA sequenced.

### Characterization of rHuN4-GM-CSF after 20 passages in MARC-145 cells

After the recombinant virus was passaged 20 times, the expression of the inserted gene was detected by IFA as described above. To investigate the growth property of the recombinant virus, MARC-145 cells cultured in 6-well plates were inoculated with an infection dose at MOI of 0.01. Culture supernatants were collected at 12, 24, 36, 48, 60, 72 and 84 hours post infection (hpi), and titrated by the method of Reed-Muench on MARC-145 cells in 96-well plates. Plaque morphology of the virus was determined by plaque assay. The recombinant rHuN4-GM-CSF virus and its corresponding parental virus were serially diluted 10-fold, and then added to MARC-145 cells in 6-well plate for 1 hour at 37°C. Plaques were stained at 37°C with crystal violet 3-5 days post infection.

### Generation of porcine bone marrow-derived dendritic cells (BMDCs)

BMDCs were isolated as described previously [34,50]. Briefly, BMHCs were harvested by flushing the bone marrow of the femur and tibia of 6-week old piglets with phosphate-buffered saline (PBS). Red blood cells were lysed with ACK lysis buffer containing 0.15 M NH4Cl, 10 mM KHCO3 and 0.1 mM Na2EDTA. BMHCs were washed twice with PBS, and cultured at 2 × 10^6^ cells/dish in 10 ml RPMI 1640 supplemented with Penicillin (100 U/ml, Gibco), Streptomycin (100 mg/ml, Gibco), L-glutamin (2 mM, Sigma), 2-mercaptoethanol (50 mM, Sigma), 10% heat-inactivated FBS (Gibco) and GM-CSF (20 ng/ml, R&D systems). After three days, another 10 ml of complete culture medium containing 20 ng/mL GM-CSF was added. At one week, non-adherent cells were collected.

### Flow cytometric analysis

Phenotypic analysis was performed by direct immunofluorescence staining in FACS buffer (DPBS containing 3% heat-inactivated FBS and 0.09% NaN_3_). Single-cell suspensions were stained with anti-CTLA-4-PE (Ancell), anti-MHC I-FITC (SLA CLASS I, Serotec) and anti-MHC II-FITC (SLA CLASS II, Serotec) in FACS buffer for 45 minutes on ice, and then washed twice with FACS buffer. The cells were analyzed on a Beckman FC500 flow cytometer with FlowJo software (TreeStar, San Carlos, CA).

### Quantification of Cytokines by RT-qPCR and Multiplex Immunoassays

To analyze the characteristics of BMDCs, cytokine assays were performed using ProcartaPlexTM Multiplex Immunoassays (Affymetrix, eBioscience) according to the manufacturer’s instructions. Briefly, BMDCs were incubated with rHuN4-GM-CSF or the parental virus at MOI of 0.3, and the supernatant was harvested at 24, 48, 72 and 96 hpi. The collected supernatants were incubated with Antibody Magnetic Beads in the individual wells of a 96-well plate for overnight. Each sample was plated in duplicate. Next, 25 μl of Detection Antibody was added to each well and then the plate was incubated for a further 30 min. Streptavidin Phycoerythrin solution was added to each well (50 μl per well). After 30 minutes incubation, the plate was read by a Luminex xMAP 200 analyzer (Luminex Corporation). The cytokine concentrations were extrapolated from standard curves using Xponent software (Luminex Corporation).

At the same time, BMDCs were collected at the indicated time points post infection for mRNA extraction using the RNeasy Mini kit (Qiagen, Hilden, Germany). cDNA was synthesized with Oligo (dT)_18_ primers and ReverAid First Strand cDNA Synthesis kit (Thermo scientific) and quantified by real-time(RT) SYBR green PCR assay was carried out with a StepOnePlus (Life Technologies, USA). The gene expression profile for cytokines, GM-CSF, IL-1β, IL-4, IL-12, IFN-γ and TNF-α were quantified and normalized with the housekeeping gene GAPDH. The primers used for these assays are described in Table 2.

**Table 2:** **Primers uesd for amplifying cytokines**

**Gene**	**Upper primer (5′–3′)**	**Lower primer (5′–3′)**
**GAPDH**	ATG GTG AAG GTC GGA GTG AAC	CGT GGG TGG AAT CAT ACT GG
**GM-CSF**	AAG CCC TGA GCC TTC TAA ACA	GGT CAA ACA TTT CAC AGA CGA
**IL-1β**	GCC CCA AAG AGA TGA AGT GCT	TCC ACT GCC ACG ATG ACA GA
**IL-4**	GCA CAT CTA CAG ACA CCA CAC	CTT TAG CCT TTC CAA GAA GTC
**IL-12**	GCC TGC TTA CCA CTT GAA CT	GCA CAG GGT TGT CAT AAA AGA G
**IFN-γ**	GCT CTG GGA AAC TGA ATG ACT	TGA CTT CTC TTC CGC TTT CTT A
**TNF-α**	ACC ACG CTC TTC TGC CTA CT	GGC TTT GAC ATT GGC TAC AAC

### Statistical analysis

Figures and statistical analyses were completed with GraphPad Prism V.5 software (GraphPad Software, San Diego, California, USA). The statistical significance of the differences among group values was determined using one-way analysis of variance (ANOVA).
